# Longitudinal Comparison of Antibiotic Resistance in Diarrheagenic and Non-pathogenic *Escherichia coli* from Young Tanzanian Children

**DOI:** 10.3389/fmicb.2016.01420

**Published:** 2016-09-07

**Authors:** Jessica C. Seidman, Lashaunda B. Johnson, Joshua Levens, Harran Mkocha, Beatriz Muñoz, Ellen K. Silbergeld, Sheila K. West, Christian L. Coles

**Affiliations:** ^1^Division of International Epidemiology and Population Studies, Fogarty International Center, National Institutes of Health, Bethesda, MDUSA; ^2^Biology Department, Morgan State University, Baltimore, MDUSA; ^3^CTS Global, Dar es SalaamTanzania; ^4^Kongwa Trachoma Project, KongwaTanzania; ^5^Dana Center for Preventive Ophthalmology, Wilmer Eye Institute, Johns Hopkins University, Baltimore, MDUSA; ^6^Department of Environmental Health, Johns Hopkins Bloomberg School of Public Health, Baltimore, MDUSA; ^7^Department of International Health, Johns Hopkins Bloomberg School of Public Health, Baltimore, MDUSA

**Keywords:** antibiotic resistance, diarrheagenic *E. coli*, Tanzania, non-pathogenic *E. coli*, children

## Abstract

Enteroaggregative, enteropathogenic, and enterotoxigenic *Escherichia coli* contribute significantly to the burden of diarrheal infections particularly in developing countries. Antibiotic resistance is increasingly common among bacterial pathogens including pathogenic *E. coli*. We assessed the relationship between pathogenic *E. coli* carriage and resistance to six antibiotics in *E. coli* isolated from young children in rural Tanzania. We surveyed temporal stability in antibiotic resistance in 2492 *E. coli* isolated from fecal samples obtained from young children in rural Tanzania collected over a 6 months period. Approximately half of the 377 children sampled were exposed to an azithromycin mass treatment program for trachoma control and half resided in control villages. Children were sampled at baseline, 1-, 3-, and 6 months following azithromycin treatment. We compared resistance to six antibiotics in pathogenic and non-pathogenic strains at the population level, within fecal specimens, and within individuals over time using chi-square tests, paired odds ratios, and logistic regression, respectively. Resistance to ampicillin and trimethoprim/sulfamethoxazole was highly prevalent (>65%). Resistance to 5 of 6 antibiotics tested and multi-drug resistance occurred more frequently in pathogenic isolates (*p* ≤ 0.001) within fecal specimens and overall. Azithromycin mass treatment exposure was significantly associated with increased odds of carriage of isolates resistant to erythromycin (OR 3.64, *p* < 0.001) and trimethoprim/sulfamethoxazole (OR 1.60, *p* < 0.05). Pathogenic isolates were approximately twice as likely to be resistant to erythromycin, ampicillin, or trimethoprim/sulfamethoxazole compared to non-pathogenic isolates from the same fecal specimen. The potential linkage between resistance and virulence in *E. coli* suggests hygiene and sanitation interventions aimed at reducing disease burden could play a role in controlling transmission of antibiotic resistance.

## Introduction

Diarrhea is estimated to be a leading cause of death globally among children under 5 years of age (Global Burden of Disease Pediatrics Collaboration et al., 2016). Approximately half of these deaths occur in sub-Saharan Africa. Although great strides have been made in reducing mortality, due in part to implementation of oral rehydration therapy and appropriate feeding practices during illness, enteric infections are still a significant cause of morbidity worldwide (Fischer Walker et al., 2012). The burden is heaviest on pre-school age children who suffer growth faltering and delayed cognitive development (Niehaus et al., 2002; Lorntz et al., 2006).

Collectively, diarrheagenic *Escherichia coli* (DEC) are among the most the most frequently identified causes of both acute and moderate-to-severe diarrhea in both community and clinical settings in low income countries (Kotloff et al., 2013; Platts-Mills et al., 2015). DEC are identified by specific virulence factors capable of causing a broad array of gastrointestinal illness. Enteroaggregative *E. coli* (EAEC), enteropathogenic *E. coli* (EPEC), and enterotoxigenic *E. coli* (ETEC) are responsible for the majority of DEC diarrheal infections in Tanzania (Vargas et al., 2004; Moyo et al., 2007; Platts-Mills et al., 2015).

Antibiotic resistant enteric pathogens are increasingly recognized in Africa (Okeke et al., 2007). Resistance prevalence among DEC isolates varies widely between sampling locations, but high levels of resistance to older, first-line antibiotics such as penicillins, and trimethoprim-sulfmethoxazole are common (Vila et al., 1999; Bii et al., 2005; Okeke et al., 2007; Moyo et al., 2011). Multi-drug resistance is rapidly becoming the norm as transmissible genetic elements, including plasmids and integrons contain multigene cassettes including genes encoding resistance to different antibiotic classes (Tellevik et al., 2007; Ajiboye et al., 2009). Resistance is also common in ubiquitous non-pathogenic *E. coli*; surveys of non-pathogenic *E. coli* have identified similar trends of highly prevalent resistance to older antibiotics (Nys et al., 2004; Djie-Maletz et al., 2008).

Relatively few studies have examined the duration of resistance in commensal flora within individuals. A study of adults visiting general practitioners in Germany found that over a 14 days period, resistance levels were stable in those not taking antibiotics, patients receiving beta-lactams showed a transient increase in resistance to ampicillin, doxycycline, and amoxicillin/clavulanic acid, and those taking doxycycline, cotrimoxazole and fluoroquinolones showed more sustained increases in resistance levels (Raum et al., 2008). A year long study of elderly residents in a long-term care facility found the median duration of colonization with multidrug-resistant *E. coli* was 178 days and co-colonization with other multidrug resistant gram negative species was common (O’Fallon et al., 2009). Karami et al. (2008) followed the colonization dynamics of ampicillin-resistant *E. coli* in infants; they found that resistant strains were able to become well established in the microbiota, persist as well as susceptible strains, and maintain colonization over a 1 year period even in the absence of direct selective pressure.

Two studies in developing country settings have also demonstrated sustained carriage of resistant commensal flora. Persistent shedding for 7 weeks or longer of ampicillin, trimethoprim or tetracycline resistant *E. coli* was highly prevalent in a 13-week study of healthy Mexican children under 2 years of age (Calva et al., 1996). A study in Tanzania of trimethoprim-sulfamethoxazole (SXT) prophylaxis for HIV-infected adults found the odds of carriage of SXT-resistant *E. coli* were significantly increased at weeks 1, 2, and 4 compared to baseline (Morpeth et al., 2008).

We investigated the relationship between DEC carriage and resistance to six antibiotics in *E. coli* isolated from young children in rural Tanzania half of whom were exposed to mass drug treatment with azithromycin (MDA) in the context of a trachoma elimination program. We previously showed that MDA exposure was associated with increased carriage of azithromycin-resistant *E. coli* in the 6 months following treatment (Seidman et al., 2014). Here we report on the concordance of resistance phenotypes between pathogenic and non-pathogenic isolates within fecal specimens and temporal stability of resistance status within individuals. We also assessed the impact of exposure to antibiotics (MDA or therapeutic) on the subsequent carriage of resistance to antibiotics of the same or different class.

## Materials and Methods

### Study Population

This study was nested within the PRET+ study, a longitudinal cohort study of the ancillary benefits of mass treatment with azithromycin for trachoma elimination (Coles et al., 2011, 2012). PRET+ was conducted in eight villages in Kongwa District, Tanzania during January–July 2009. Four villages received MDA as part of a trachoma control program. For ethical reasons, villages in the same geographic area with trachoma rates below the intervention threshold were selected as controls. The sub-cohort sampled in this study was drawn from both intervention and control villages. Household enumeration occurred during a census in December 2008; households with at least one resident <5 years of age were eligible. In each village, one child <5 was randomly selected for each of 130 randomly selected households for the PRET+ cohort. Children <3 years were eligible for the *E. coli* sub-cohort. A total of 40 children in each village attending the baseline survey were selected for the *E. coli* sub-cohort.

### Ethical Review and Consent Procedures

The PRET+ study and the nested *E. coli* study were approved by the Committee for Human Subjects Research of the Johns Hopkins School of Medicine and the Tanzanian National Institute for Medical Research. Prior to the initiation of the PRET+ study, meetings were held with community leaders to explain the goals of the study and the collection protocol. Selected participants were invited to attend the baseline survey in January 2009. Upon arrival at the survey, participants were asked to provide their consent to be in the PRET+ cohort. Participants were asked to sign a written consent form (translated into Swahili) detailing PRET+ study procedures. The content of the consent form was also verbally communicated to all participants by study personnel trained to administer consent. Literate participants signed the consent form; illiterate participants indicated consent by providing a thumbprint. A parent or caregiver provided consent on behalf of each child in the PRET+. After proceeding through the questionnaire/specimen collection stations of the survey, parents/caregivers of children eligible to be in the *E. coli* cohort had the goals of the *E. coli* study explained and provided verbal consent. Subjects in the both the PRET+ and *E. coli* cohorts were allowed to refuse participation at any time during the 6 months PRET+ study without penalty.

### Data and Specimen Collection

Sequential cross-sectional surveys to assess prevalence of diarrheal and other symptoms were conducted at baseline (2 days prior to MDA in the intervention communities), and 1-, 3-, and 6-months post-MDA. The MDA regimen consisted of a single dose of azithromycin (20 mg/kg) administered to all village residents except children <6 months of age and pregnant women. During surveys, mothers/caregivers of children in the PRET+ cohort were asked about presence of diarrheal symptoms (defined as ≥3 loose or watery stools in a 24-h period). Rectal swabs were collected from *E. coli* sub-cohort children during cross-sectional surveys. Swabs were placed immediately into Cary-Blair and stored at 4°C once they reached the study office.

Households were visited twice weekly to ascertain incidence of diarrheal and other symptoms via a standardized questionnaire. Diarrheal stool samples were collected from all children in the PRET+ cohort reporting symptoms during household illness surveillance visits, regardless of whether they were in the *E. coli* sub-cohort sampled during the cross-sectional surveys. Diarrheal stool collection occurred during the first 4 months of the study period. Symptomatic children in all study villages were treated with amoxicillin for acute respiratory and ear infections and chloramphenicol for bloody diarrhea. Rectal swabs and diarrheal stool specimens were transported to the Kongwa District Hospital laboratory for isolation and archiving of *E. coli* and then shipped to Johns Hopkins University, USA for further analysis.

Medical dispensaries and shops were surveyed regarding locally available antibiotics: penicillin/penicillin derivatives were available in seven villages; cotrimoxazole and erythromycin in 5; chloramphenicol in 4 and ciprofloxacin in 3. Other antibiotics that were available included metronidazole (four villages), doxycycline (three villages) and ceftriaxone (one village).

### Laboratory Methods

Fecal specimens were streaked on MacConkey agar (Difco) and grown overnight at 37°C. *E. coli* were presumptively identified on the basis of lactose fermentation (LF) and colony morphology. Three LF-positive colonies were inoculated into nutrient agar stabs and grown overnight at 37°C followed by storage at room temperature. Isolates were considered *E. coli* if LF-positive, indole-positive (DMACA Indole Reagent droppers, BD), and citrate-negative (Simmons citrate agar slants, BD). *E. coli* isolates were inoculated into Luria broth, grown overnight at 37°C with shaking, then frozen at -80°C with 10% glycerol.

*Escherichia coli* isolates were tested for susceptibility to ampicillin (AMP) (10 μg), amoxicillin-clavulanic acid (AMC) (20/10 μg), chloramphenicol (CHL) (30 μg), ciprofloxacin (CIP) (5 μg), erythromycin (ERY) (15 μg), and trimethoprim-sulfamethoxazole (SXT) (1.25/23.75 μg) (BBL Sensi-Discs, BD) using the disk diffusion method on Mueller-Hinton agar (Difco) (Bauer et al., 1966). Zone diameters (ZD) were measured in millimeters. Isolates were classified as susceptible or resistant according to CLSI guidelines (intermediate zones were classified resistant) (Clinical Laboratory Standards Insitute, 2008). Since there are no guidelines for macrolide susceptibility breakpoints for *E. coli*, a conservative definition was used whereby an isolate was classified as ERY resistant only if there was no growth inhibition (i.e., ZD = 0 mm) (Seidman et al., 2014). Multi-drug resistance was defined as resistance to ≥3 antibiotics. *E. coli* ATCC 25922, *Staphylococcus aureus* ATCC 25923, and *S. aureus* 29213 were used for quality control testing of antibiotic reagents.

Diarrheagenic *E. coli* strains were identified using a multiplex polymerase chain reaction (PCR) assay with primers to detect the genes *eltB* and *estA* for ETEC, *eae* and *bfpA* for EPEC, *aaiC* and *aatA* for EAEC (Supplementary Table S1) (Lüscher and Altwegg, 1994; Schmidt et al., 1995; Oswald et al., 2000; Nguyen et al., 2005; Boisen et al., 2008; Taniuchi et al., 2012). Initial reactions were conducted on pools of isolates from the same fecal specimen. All isolates from negative pools were classified as non-pathogenic; PCR was repeated on individual isolates from positive pools to distinguish pathotypes for each isolate in the pool. Approximately 10 μl of frozen culture was suspended in 50 μl of sterile water then boiled for 10 min; 3 μl of the supernatant was used as the template. The assay was conducted in a 20 μl reaction containing 3 μl of template, 20 μM of each primer, 2.5 μl of 10X Thermopol Buffer (with 2 mM MgCl_2_) (New England Biolabs), 2 μl 10 mM dNTP mix (Fermentas), 0.25 μl Taq DNA polymerase (NEB), and 7.37 μl molecular grade water. Thermal conditions were as follows: initial denaturation at 96°C for 4min, 35 cycles of denaturation at 95°C for 20 s, annealing at 57°C for 20 s, elongation at 72°C for 1 min, and final extension at 72°C for 7 min. Reactions were performed in 96-well plates in an Eppendorf Mastercycler Gradient PCR machine. Electrophoresis and UV translumination were used to separate and visualize PCR products [2% agarose gel in TBE buffer + ethidium bromide (1 μg/ml)]. All runs included positive and negative controls. Pathogenic isolates were classified as follows: ETEC: LT+ and/or ST+; EPEC: *eae*+ or *eae*+/*bfp*A+; EAEC: *aaiC*+ and/or *aatA*+. ETEC H10407, EPEC 2348/11 and EAEC 042 strains were used as positive controls (PCR protocol and control strains courtesy of J.P. Nataro).

### Statistical Analysis

We used binary susceptible/resistant classification to calculate the proportion of isolates resistant to each individual antibiotic and to determine the frequency of co-resistance patterns. Differences in pathogenic detection in symptomatic and asymptomatic fecal specimens as well as differences in antibiotic resistance frequency among pathogenic and non-pathogenic isolates were compared using chi-squared tests. We compared the resistance status of pathogenic and non-pathogenic isolates within those individual fecal samples where ≥1 isolate was pathogenic and ≥1 isolate was non-pathogenic. We used 1000 bootstrapped samples randomly selected (with replacement) from the isolate pairs (such that each fecal sample contributed only one isolate pair to the test set) to calculate the distribution of paired odds ratios and McNemar’s test statistics for each antibiotic.

We assessed the within-person temporal relationship between DEC carriage and resistance status using first order Markov models to quantify the odds of a transition from susceptible to resistant as a function of DEC carriage and time. Models were fit separately for each antibiotic. These logistic models were formulated such that the odds of having a resistant isolate in the current specimen is a function of having a resistant isolate in the prior specimen and presence of pathogenic isolates in the current or prior specimens, adjusting for known antibiotic exposures (MDA or therapeutic amoxicillin or chloramphenicol), and length of time (days) between specimen collection. MDA exposure was defined as residence in a MDA village. Generalized estimating equations with exchangeable correlation were used to adjust for village-level clustering and robust standard errors were calculated to account for residual within-person correlation due to repeated measures (Liang and Zeger, 1986). Analyses were performed using STATA 12 (StataCorp, College Station, TX, USA).

## Results

**FIGURE 1:**
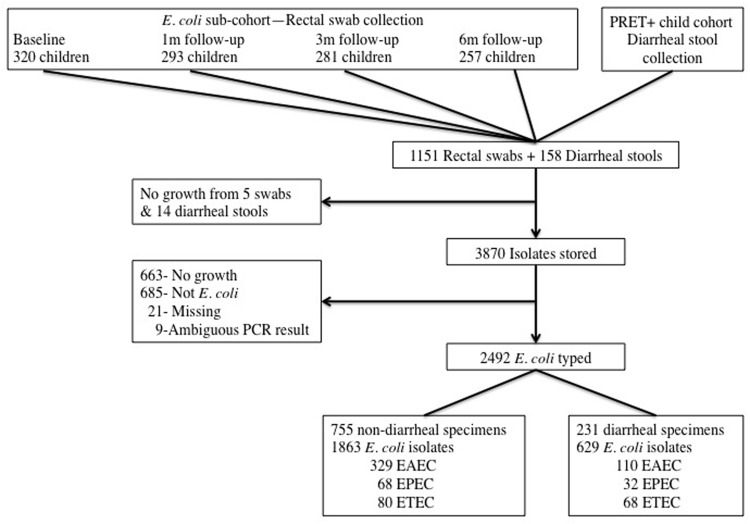
**Flow chart of specimen collection and *E. coli* isolation**.

We collected 1151 rectal swabs and 158 diarrheal stools from 377 individual children; 2492 *E. coli* isolates were recovered from these specimens (**Figure 1**). The mean age of children included in the *E. coli* sample was 1.13 years, average household size was 5.6 (standard deviation 2.1) people and 69.8% of participant households had a latrine. On average, children contributed 6.6 isolates in total (range 1–18). Approximately 25% of isolates were obtained from children reporting diarrheal symptoms (287 from rectal swabs and 342 from diarrheal stools). Nearly half of the children were MDA exposed (49.3%), 239 received therapeutic amoxicillin, and 32 received therapeutic chloramphenicol.

The majority of *E. coli* isolated were non-pathogenic (72.4%); 439 (17.6%), 100 (4.0%), and 148 (5.9%) of isolates were identified as EAEC, EPEC, and ETEC respectively. A total of 243 individual children (64.5%) had ≥1 DEC type isolated during the study. In cross-sectional survey specimens, ETEC and EAEC were detected more frequently in isolates from symptomatic children (ETEC: 7.0 % vs. 4.3%, *p* = 0.045; EAEC: 23.0% vs. 17.7%, *p* = 0.030) whereas there was no significant difference in EPEC detection (4.2% vs. 3.7%, *p* = 0.658). Amongst isolates from diarrheal stools, ETEC was identified most frequently (14.0%), followed by EAEC (12.9%) and EPEC (5.9%).

The observed prevalence of resistance to six antibiotics amongst pathogenic and non-pathogenic *E. coli* is shown in **Table 1**. Resistance to AMP and SXT was most common amongst isolates of all *E. coli* types. Resistance to these two antibiotics was particularly high among pathogenic isolates with more than 80% of DEC isolates resistant to AMP and SXT. In contrast, CIP resistance was rare, seen in only 15 isolates (all non-pathogenic). The majority of isolates (75.9%) were resistant to ≥1 antibiotic and 36.1% were multi-drug resistant. Multi-drug resistance was common in EAEC (61%) and EPEC (42%) but less frequent in ETEC (16.8%). Resistance to most of the antibiotics and multi-drug resistance occurred more frequently in DEC isolates (*p* ≤ 0.001). Resistance rates amongst pathogenic isolates from diarrheal specimens were generally similar to rates amongst pathogenic isolates from non-diarrheal specimens; the sole exception was ERY resistance which was seen more frequently in EPEC isolated from non-diarrheal specimens compared to EPEC from diarrheal specimens (41.1% vs. 18.8%, *p* = 0.027).

**Table 1:** Antibiotic resistance prevalence (%) in pathogenic and non-pathogenic E. coli*.

	All isolates (*N* = 2492)	Non-pathogenic (*N* = 1805)	Pathogenic (*N* = 687)	EAEC (*N* = 439)	EPEC (*N* = 100)	ETEC (*N* = 148)
AMC	10.6	9.2	14.4	16.4	15.0	8.1
AMP	65.1	59.1	80.9	82.9	79.0	76.4
CHL	14.3	12.0	20.5	26.7	9.0	10.1
CIP	0.6	0.8	0	0	0	0
ERY	29.4	27.2	35.4	45.3	34.0	6.8
SXT	68.3	63.1	82.0	87.5	79.0	67.6
≥3 antibiotics	36.1	31.3	48.8	61.0	42.0	16.8

*Chi-squared tests for differences in frequency of resistance comparing pathogenic and non-pathogenic isolates statistically significant (*p* < 0.001) for all antibiotics and multi-drug resistance.

A total of 177 fecal specimens yielded isolate sets that enabled within specimen comparison of resistance status between pathogenic and non-pathogenic isolates. We could not assess CIP resistance concordance because no pathogenic isolates were resistant. For all five antibiotics considered, the median odds of resistance were greater for DEC isolates: median odds ratios (2.5, 97.5 percentiles) were 1.67 (1.25, 2.27) for AMC, 1.69 (1.45, 2.00) for CHL, 1.95 (1.61, 2.33) for ERY, 2.22 (1.90, 2.71) for SXT, and 2.72 (2.30, 3.33) for AMP (Supplementary Figure S1). The associations between pathogenicity and resistance to AMP, SXT, and ERY were highly significant with median *p*-values <0.025.

We assessed how DEC carriage affected within-person temporal stability of resistance status (**Table 2**). Again, CIP was excluded due to the low resistance frequency. Detection of pathogenic isolates in the current fecal specimen was strongly associated with current resistance to AMC, AMP, ERY, and SXT (*p* < 0.02) whereas detection of pathogenic isolates in the prior specimen was not statistically significantly associated with current resistance to any antibiotic. Carriage of isolates resistant to AMC, AMP, CHL or SXT in the prior specimen was associated with increased odds of subsequent resistance; the relationship was statistically significant for AMP and CHL (*p* < 0.05). MDA exposure was significantly associated with increased odds of carriage of isolates resistant to ERY (OR 3.64, *p* < 0.001) and SXT (OR 1.60, *p* < 0.05). A detailed exploration of the impact of MDA on macrolide resistance has been published previously (Seidman et al., 2014). Exposure to therapeutic amoxicillin was not significantly associated with the odds of resistance carriage for AMP or AMC; similarly, exposure to therapeutic chloramphenicol was not associated with CHL resistance status.

**Table 2:** Logistic regression models to assess the temporal relationship between carriage of antibiotic resistance and pathogenic *E. coli*.

	AMC	AMP	CHL	ERY	SXT
Resistant isolates in previous specimen	1.36 (0.84, 2.21)	1.40 (1.05, 1.86)*	1.65 (1.02, 2.69)*	0.99 (0.58, 1.70)	1.32 (0.99, 1.76)
Pathogenic isolates in current specimen	1.76 (1.10, 2.80)*	4.52 (3.78, 5.42)***	1.79 (0.88, 3.68)	1.73 (1.10, 2.72)*	3.18 (1.59, 6.38)**
Pathogenic isolates in previous specimen	0.92 (0.63, 1.36)	0.81 (0.48, 1.36)	1.16 (0.82, 1.65)	1.00 (0.77, 1.29)	0.86 (0.69, 1.07)
Exposure to MDA	1.30 (0.82, 2.06)	1.64 (0.91, 2.94)	0.93 (0.62, 1.39)	3.64 (2.38, 5.78)***	1.60 (1.09, 2.35)*
Treatment with antibiotics since last specimen	1.00 (0.76, 1.32)†	0.79 (0.58, 1.07)†	1.06 (0.53, 2.12)§		
Days between previous specimen and current specimen	0.99 (0.99, 0.99)***	1.00 (0.99, 1.01)	1.00 (0.99, 1.01)	0.99 (0.98, 1.00)	0.99 (0.98, 1.01)

Odds ratios and 95% CI for ever positive. *p*-values indicated as : * < 0.05, ** < 0.01, *** < 0.001. †Exposure to amoxicillin. §Exposure to chloramphenicol.

We observed 28 distinct antibiotic resistance phenotypes using binary resistance categorization (Supplementary Table S2). Resistance to both AMP and SXT was the most frequently observed combination and was more common than resistance to either antibiotic alone. Joint AMP/SXT resistance occurred in 12 of the 28 unique patterns. Resistance to CIP was seen only in concert with resistance to other antibiotics. Two non-pathogenic isolates were resistant to all 6 antibiotics tested.

## Discussion

We assessed resistance to six antibiotics in pathogenic and non-pathogenic *E. coli* isolated from young children in rural Tanzania over a 6-month period. We compared the resistance prevalence by pathogenic type in the population of isolates as well as within individual children over time. Three quarters of isolates were resistant to ≥1 antibiotic and more than 30% were multi-drug resistant. Overall, AMP and SXT resistance was highly prevalent while CIP resistance was rare. These findings were similar to prior studies of *E. coli* resistance in African sites although a more recent study in Ghanaian adults reported much higher levels of quinolone resistance than we observed in our study (Nys et al., 2004; Djie-Maletz et al., 2008; Moyo et al., 2011; Namboodiri et al., 2011).

Approximately 30% of the *E. coli* isolated were ETEC, EPEC, or EAEC and resistance to 5 of 6 antibiotics occurred significantly more frequently in DEC isolates compared to non-pathogenic isolates. In contrast, a study in young Brazilian children found no differences in AMP and SXT resistance prevalence between pathogenic and non-pathogenic *E. coli* (Garcia et al., 2011). The high prevalence of resistance in the DEC strains we observed is in keeping with a previous Tanzanian study where prevalence of resistance in ETEC, EAEC and EPEC isolated from pediatric diarrhea cases was very high for AMP (83–90%), CHL (25–57%), and SXT (79–90%) (Vila et al., 1999).

Diarrheagenic *E. coli* were approximately twice as likely to be resistant to ERY, AMP, or SXT compared to non-pathogenic isolates from the same fecal specimen. We were unable to find other examples in the literature of within-person comparison of resistance phenotypes between *E. coli* pathotypes, however, some studies have found evidence for genetic linkages between resistance genes and virulence factors in *E. coli* isolated from swine (Boerlin et al., 2005; Lay et al., 2012).

At the person level, detection of AMP-, SXT-, or CHL-resistant isolates at a previous sampling time increased the odds of subsequent resistance carriage. We expected that prior resistance status would be the most significant predictor of current status, hypothesizing that once resistance was established within an individual, it would be sustained. However, although resistance status at the previous time point was associated with current status, isolation of DEC strains from the current specimen was a greater predictor of current resistance status for AMC, AMP, ERY, and SXT than status at the previous time point. While resistance was maintained within individuals, this may reflect continual acquisition of resistant strains from the environment rather than long-term carriage of a clonal strain. A study in adults returning to Australia following international travel did find a high rate of clonal carriage of resistant *E. coli* for 2 months or longer, however, the risk of environmental exposures and frequency of acquisition of resistant strains may be considerably lower in adults living in Australia compared to our study population of young children in rural Tanzania (Rogers et al., 2012). It is a limitation of our study that we could not distinguish between long-term carriage of resistant strains versus new colonization with phenotypically similar strains.

Direct exposure to antibiotic therapy is thought to a major driver of resistance. In our study, MDA exposure was highly associated with increased ERY resistance but exposure to therapeutic amoxicillin and chloramphenicol was not associated with subsequent AMP or CHL resistance carriage. Studies of post-MDA macrolide resistance in *S. pneumoniae* have found a range of resistance levels from 0 to 82% (Leach et al., 1997; Fry et al., 2002; Haug et al., 2010; Coles et al., 2013). Previously we showed previously that macrolide resistance in *E. coli* increased significantly following MDA then tapered over the subsequent 6-month period, although it remained elevated above baseline levels (Seidman et al., 2014). In the four treatment villages, the entire community was treated simultaneously during MDA, thus the selective pressure on the bacterial community was substantial. In contrast, in both intervention and control villages, therapeutic antibiotics were given to individuals at different times and likely did not affect the bacterial population in the same way. Prior studies have shown a relationship between recent therapeutic antibiotic use and carriage of resistant *E. coli*, but the effects may be short lived (Stürmer et al., 2004; Lietzau et al., 2007; Samore et al., 2011). In our study, the time between antibiotic therapy and specimen collection varied, which may have limited our ability to detect transient increases in resistance. Since AMP resistance carriage was already highly prevalent at baseline, the impact of amoxicillin therapy on the development of AMP resistance may have been difficult to observe. We chose to assess resistance to commonly used, locally available antibiotics, thus we did not characterize the prevalence of cephalosporin resistance; this would be a valuable area for future research as extended-spectrum beta-lactamases become more wide-spread in around the world (Woerther et al., 2013).

We found significant within-person heterogeneity in resistance status over time. Since we were only able to test up to three isolates per fecal specimen and our multiplex PCR assay detected only three pathogenic types of DEC, we may not have sampled the full heterogeneity of resistance patterns within individual specimens and some isolates we considered non-pathogenic may have been DEC pathotypes not detected by our assay. Previous studies of DEC in Tanzania found EAEC, EPEC, and ETEC to be most common; studies screening for more DEC types have detected enterohemorrhagic and enteroinvasive *E. coli* at very low frequencies (Vila et al., 1999; Gascon et al., 2000; Vargas et al., 2004; Moyo et al., 2007). We did not identify genes encoding resistance. However, given that our results showed a higher prevalence of resistance among DEC strains, it is crucial that future studies evaluate differences in resistance mechanisms between pathogenic and non-pathogenic *E. coli* as well as characterize the mobility of these genetic elements to understand transmission within the microbial community.

We found significant antibiotic resistance levels in pathogenic *E. coli* isolated from young Tanzanian children. Specific multi-drug resistance phenotypes were common to both pathogenic and non-pathogenic strains, indicating that commensal *E. coli* can serve as a sentinel organism for patterns in circulating pathogenic strains. Resistance was more frequent in DEC isolates compared to non-pathogenic isolates at both individual and population scales. The potential linkage between resistance genes and virulence determinants underscores the urgent need for a renewed focus on implementation of sanitation and hygiene interventions in low-income countries. Children in these settings are continuously exposed enteropathogens from contaminated home environments (Mattioli et al., 2014). Sanitation interventions may serve a dual purpose in limiting both enteric infection and an environmental reservoir of antimicrobial resistance thus mitigating the burden of a significant proportion of several pediatric infectious diseases.

## Author Contributions

JS and CC designed the study in collaboration with SW, BM, and HM. JS conducted the fieldwork with assistance from HM and JL. JS and LJ conducted the laboratory work; JS conducted the statistical analysis and drafted the manuscript. CC and ES contributed to the interpretation of the data. All authors contributed to the manuscript editing and approved the final manuscript.

## Conflict of Interest Statement

The authors declare that the research was conducted in the absence of any commercial or financial relationships that could be construed as a potential conflict of interest.
