# Supplementary material for: Longitudinal Comparison of Antibiotic Resistance in Diarrheagenic and Non-pathogenic Escherichia coli from Young Tanzanian Children
Source: Front Microbiol. 2016 Sep 7;7:1420. doi: 10.3389/fmicb.2016.01420 (PMC5013055; doi:10.3389/fmicb.2016.01420)
Supplement: Supplementary file 1 [file Image_1.PDF]

Supplementary Figure 1. Distribution of bootstrapped paired odds ratios and McNemar's p values for the association between antibiotic resistance and pathogenicity in *E. coli* isolated from the same fecal specimen

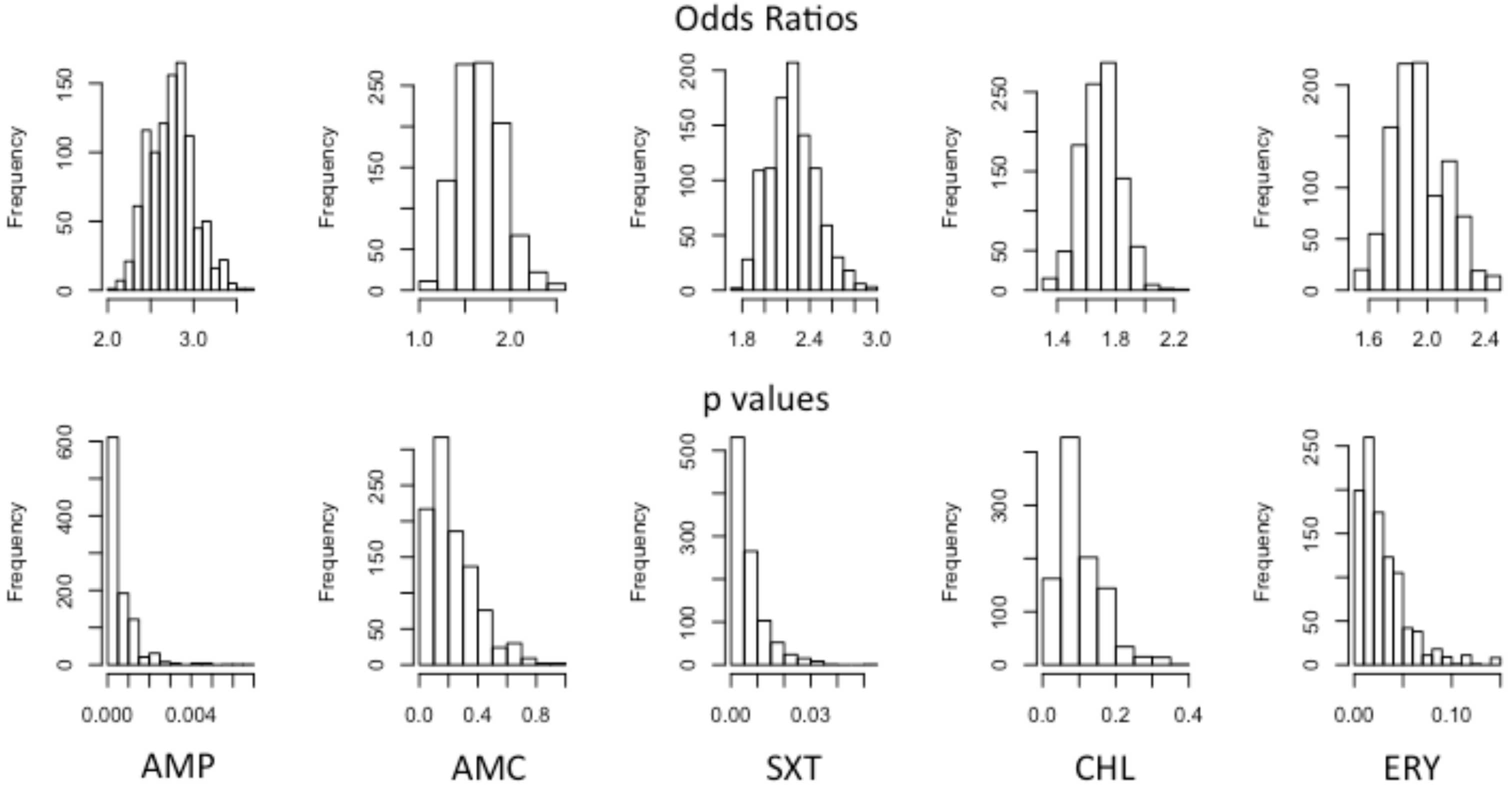

Supplementary Figure S1. Distribution of bootstrapped paired odds ratios and McNemar's p values for the association between antibiotic resistance and pathogenicity in *E. coli* isolated from the same fecal specimen. AMP=Ampicillin, AMC=Amoxicillin-Clavulanic Acid, SXT=Trimethoprim-Sulfamethoxazole, CHL=Chloramphenicol, ERY=Erythromycin
