# Supplementary material for: Longitudinal Comparison of Antibiotic Resistance in Diarrheagenic and Non-pathogenic Escherichia coli from Young Tanzanian Children
Source: Front Microbiol. 2016 Sep 7;7:1420. doi: 10.3389/fmicb.2016.01420 (PMC5013055; doi:10.3389/fmicb.2016.01420)
Supplement: Supplementary file 2 [file Table_1.DOCX]

**Supplementary Table 1 Primer sequences for ETEC, EAEC, and EPEC amplification**

| ***E. coli* Type** | **Primer Name** | **Target Gene** | **Citation** | **Primer Sequence** | **Amplimer size (bp)** |
| --- | --- | --- | --- | --- | --- |
| ETEC | LT_B_ | *eltB* | Taniuchi 2012 | **5′** CACACGGAGCTCCTCAGTC **3′**  **5′** CCCCCAGCCTAGCTTAGTTT **3′** | 508 |
|  | ST | *estA* | Nguyen 2005 | **5′** GCTAAACCAGTA^G^_A_GGTCTTCAAAA **3′**  **5′** CCCGGTACA^G^_A_GCAGGATTACAACA **3′** | 147 |
| EAEC | aaiC | *aaiC* | Boisen 2008 | **5′** ATTGTCCTCAGGCATTTCAC **3′**  **5′** ACGACACCCCTGATAAACAA **3′** | 215 |
|  | CVD432 | *aatA* | Schmidt 1995 | **5′** CTGGCGAAAGACTGTATCAT **3′**  **5′** CAATGTATAGAAATCCGCTGTT **3′** | 630 |
| EPEC | eae | *eae* | Oswald 2000/ Luscher 1994 | **5′** CCCGAATTCGGCACAAGCATAAGC **3′**  **5′** CCCGGATCCGTCTCGCCAGTATTCG **3′** | 881 |
|  | bfpA | *bfpA* | Taniuchi 2012 | **5′** GGAAGTCAAATTCATGGGGG **3′**  **5′** GGAATCAGACGCAGACTGGT **3′** | 300 |
