# Supplementary material for: Longitudinal Comparison of Antibiotic Resistance in Diarrheagenic and Non-pathogenic Escherichia coli from Young Tanzanian Children
Source: Front Microbiol. 2016 Sep 7;7:1420. doi: 10.3389/fmicb.2016.01420 (PMC5013055; doi:10.3389/fmicb.2016.01420)
Supplement: Supplementary file 3 [file Table_2.DOCX]

**Supplementary Table 2. Antibiotic resistance patterns of pathogenic and non-pathogenic *E. coli* isolates***

| **Resistance classification**** | **All *E. coli*** | **Non-pathogenic** | **Pathogenic** | **EAEC** | **EPEC** | **ETEC** |
| --- | --- | --- | --- | --- | --- | --- |
| N | 2492 | 1805 | 687 | 439 | 100 | 148 |
| All susceptible | 601 (24.1) | 531 (29.4) | 70 (10.2) | 25 (5.7) | 14 (14.0) | 31 (20.9) |
| AMP SXT | 570 (22.9) | 393 (21.8) | 177 (25.8) | 73 (16.6) | 31 (31.0) | 73 (49.3) |
| AMP SXT ERY | 420 (16.9) | 277 (15.3) | 143 (20.8) | 120 (27.3) | 20 (20.0) | 3 (2.0) |
| SXT | 226 (9.1) | 170 (9.4) | 56 (8.2) | 46 (10.5) | 6 (6.0) | 4 (2.7) |
| AMP SXT CHL | 141 (5.7) | 67 (3.7) | 74 (10.8) | 54 (12.3) | 8 (8.0) | 12 (8.1) |
| AMP SXT AMC ERY | 100 (4.0) | 61 (3.4) | 39 (5.7) | 26 (5.9) | 11 (11.0) | 2 (1.4) |
| AMP | 86 (3.5) | 61 (3.4) | 27 (3.9) | 14 (3.2) | 3 (3.0) | 10 (6.8) |
| AMP SXT ERY CHL | 80 (3.2) | 53 (2.9) | 27 (3.9) | 26 (5.9) | 1 (1.0) |  |
| AMP SXT AMC ERY CHL | 55 (2.2) | 35 (1.9) | 20 (2.9) | 19 (4.3) |  | 1 (0.7) |
| AMP SXT AMC | 41 (1.6) | 29 (1.6) | 12 (1.7) | 6 (1.4) | 2 (2.0) | 4 (2.7) |
| AMP ERY | 40 (1.6) | 33 (1.8) | 7 (1.0) | 3 (0.7) | 2 (2.0) | 2 (1.4) |
| AMP SXT AMC CHL | 31 (1.2) | 17 (0.9) | 14 (2.0) | 13 (3.0) |  | 1 (0.7) |
| SXT CHL | 19 (0.8) | 18 (1.0) | 1 (0.1) | 1 (0.2) |  |  |
| AMP AMC | 14 (0.6) | 7 (0.4) | 7 (1.0) | 4 (0.9) | 1 (1.0) | 2 (1.4) |
| AMP CHL | 11 (0.4) | 8 (0.4) | 3 (0.4) | 2 (0.5) |  | 1 (0.7) |
| CHL | 11 (0.4) | 9 (0.5) | 2 (0.3) | 2 (0.5) |  |  |
| AMP AMC ERY | 11 (0.4) | 5 (0.3) | 6 (0.9) | 4 (0.9) |  | 2 (1.4) |
| AMP SXT ERY CIP | 10 (0.4) | 10 (0.6) |  |  |  |  |
| ERY | 5 (0.2) | 4 (0.2) | 1 (0.1) | 1 (0.2) |  |  |
| AMP AMC CHL | 4 (0.2) | 4 (0.2) |  |  |  |  |
| SXT ERY | 3 (0.1) | 3 (0.2) |  |  |  |  |
| AMC | 2 (0.1) | 1 (0.1) | 1 (0.1) |  | 1 (1.0) |  |
| AMC ERY | 2 (0.1) | 2 (0.1) |  |  |  |  |
| AMP SXT AMC ERY CHL CIP | 2 (0.1) | 2 (0.1) |  |  |  |  |
| AMP SXT AMC ERY CIP | 2 (0.1) | 2 (0.1) |  |  |  |  |
| SXT ERY CHL | 1 (0.0) | 1 (0.1) |  |  |  |  |
| AMP SXT ERY CHL CIP | 1 (0.0) | 1 (0.1) |  |  |  |  |
| AMP AMC ERY CHL | 1 (0.0) | 1 (0.1) |  |  |  |  |

*Counts of isolates; numbers in parentheses represent column percents.

**Binary resistance classification based on CLSI breakpoints; ERY resistance defined as complete lack of growth inhibition (*ie* zone diameter=0).
